# Special Issue: Mycorrhizal Fungi in Sensitive Environments

**DOI:** 10.3390/jof1020168

**Published:** 2015-08-10

**Authors:** Francesca Scandellari

**Affiliations:** Francesca Scandellari, Free University of Bolzano-Bozen Piazza Università 5, Bolzano-Bozen I-39100, Italy; E-Mail: Francesca.Scandellari@unibz.it; Tel.: +39-471-017809; Fax: +39-471-017009.

**Keywords:** glaciers, mountain, ecotone, nitrogen, ecosystem protection and restoration

## Abstract

The scope of this special issue is to understand whether and how mycorrhizal symbiosis can be included as an agriculture and agroforestry tool that promotes more environmentally friendly practices, and whether it promotes the protection of sensitive areas. Three papers are included in this special issue, each dealing with a different sensitive environment. These papers present fundamental aspects that should be taken into account when planning or reporting studies related to mycorrhizal fungi in sensitive environments.

With this special issue, we intended to address the question of whether (and how) improving and protecting mycorrhizal symbiosis can promote more sustainable agricultural and agroforestry practices and support the protection of sensitive areas.

Because of their high genetic and physiological diversity, mycorrhizal fungi are involved in many biogeochemical cycles [1] with the most studied being carbon [2], nitrogen [3,4], phosphorous [5] and water [6,7]. In addition, mycorrhizal fungi are amply widespread since they can be found in the roots of most plant species [1]. These characteristics make mycorrhizal fungi a potential powerful tool to be used in actions towards a sustainable management of the environment [8,9,10]. For example, the potential of mycorrhizal fungi to contribute to the ecosystem restoration after disturbances has been studied in several ecosystems [11], such as in relation to mining aftermath [12] or in sensitive environments such as riparian zones [13]. In agricultural ecosystems, adding mycorrhizal inocula to improve soil and plant health without the application of expensive and potentially polluting chemicals has become more popular [14].

These new opportunities opened up by mycorrhizal research are especially interesting for those areas of the world particularly sensitive to environmental disruption due to inadequate soil management and to climate change. Areas of this kind are distributed throughout the world and found in many different habitats. Mountain areas include many of these sensitive areas, and therefore they require special care to avoid the excessive exploitation of resources, such as water and biodiversity, and to limit environmental pollution due to, for example, nitrogen leaching into waters [15]. This topic is of particular importance because of the many people relying on mountain resources both directly, through crop production and animal farming, and indirectly, for example as consumers of downstream water [15]. Particularly sensitive to climate change are also glaciers, whose area has noticeably decreased since the end of the Little Ice Age in the second half of the 19th century [16]. Concern related to the loss of permanent glaciated areas, both at high elevations and at the Poles, is a hot topic of modern research and politics. The exposure of new soil due to ice melting might be considered an extreme example of land use change. More generally, the transitional areas between different biomes, the ecotones, can also be listed as sensitive environments [17].

The papers submitted to this special issue of the *Journal of Fungi* (*JoF*) present fundamental aspects to be taken into account when planning or reporting studies related to mycorrhizal fungi in sensitive environments.

Jumponnen and colleagues highlighted the importance of integrating different techniques to more properly represent the high complexity of sensitive environments [18]. In many cases and for many reasons––money issues, time available or labor needed—the choice of using a single technique is favored. However, as demonstrated by this paper, even the most advanced techniques may fail to detect important aspects in the studied system. The authors used four different techniques, namely sporocarp surveys, ectomycorrhizal root-tip analysis and next generation sequencing of soil fungi using ITS and LSU, to compare the ECM diversity in a sensitive environment such as a glacier forefront. Despite the expected low richness of species—because of the extreme environment that was studied—the four techniques revealed an interesting and unexpected scenario. The core of the study however is that despite some overlap among the data, there were significant differences among the results obtained with the four techniques. The reasons for this mismatch can only be guessed at this point, and include the patchiness of the ectomycorrhizal fungi genetics, PCR primer bias, or temporal differences due to different sampling methods being carried out at different times. This issue is highly relevant in environmental studies and, as highlighted by Jumponnen and colleagues, it is clear that there is a need to use more than one technique and to interpret data based on the comparison among their results.

The study of Rainer and colleagues deals with the restoration of habitats that have been deforested for a long time [19]. The main point is that mycorrhizal inocula are lost after a certain amount of time, therefore successful reforestation strategies have to take into account not only the vegetation but also the relative mycorrhizal species. Failing to do this would lead to a failure in the restoration activity. Another point to take into account is to find the proper mycorrhizal species. Indeed, a successful inoculation would last several years and would sustain the growth of tree species, while inappropriate species would soon be replaced with loss of time and resources of the environment, and only under the condition that suitable species are already present on site. These authors also approached the study using different techniques, namely mycorrhizal roots morphotyping, ITS-RFLP profiles of tips, sequencing, ergosterol extraction and hyphal length. This study demonstrates the importance of using different techniques to obtain coherent results. In this study, the authors detected no differences in ECM communities due to the effect of environmental altered temperature and antropic nitrogen fertilization influence. Moreover, N fertilization did not modify ECM composition, but it caused a reduction in fungal biomass.

Rua and colleagues focused their study on a transitional area between a mature forest and an open field being colonized by young conifer trees [20]. The effect of this land use change is determined on the ectomycorrhizal community composition and activity through the study of extracellular enzymatic activity. The results of this study indicate that many environmental variables may affect the composition and the activity of the mycorrhizal community, but not necessarily both. Therefore, it becomes clear that when conducting studies of this kind, the mere knowledge of the community structure might be of some use, but the similarities of composition do not necessarily imply a similar function. Although this study was limited to differences at the Family level—and therefore, as also the authors underline, the possible contribution of different species might be missing—it provides supporting evidences on how soil composition and environmental factors might be incorporated into models to predict fungal structure and activity.

This special issue presents several aspects related to the role of mycorrhizal fungi in agroforestry and to the analytical methods available to study these environments. This collection of papers offers a glimpse of the potential of mycorrhizal research to restoration and of the effect of land use change on the activity of mycorrhizal communities.

To conclude, I would like to thank each of the authors for their contribution and support in producing this special issue on Mycorrhizal Fungi in Sensitive Environments. Finally, I wish also to acknowledge the thorough work of the peer reviewers who contributed to improve the submitted manuscripts and who, with their comments to the manuscripts, provided useful suggestions to write this Editorial. I also thank the staff members of the *JoF* Editorial Office, in particular Ms. Qi Yan, for their support.
